# C-peptide enhances glucagon secretion in response to hyperinsulinemia under euglycemic and hypoglycemic conditions

**DOI:** 10.1172/jci.insight.148997

**Published:** 2021-06-22

**Authors:** Mary Courtney Moore, Shana O. Warner, Yufei Dai, Nicole Sheanon, Marta Smith, Ben Farmer, Rebecca L. Cason, Alan D. Cherrington, Jason J. Winnick

**Affiliations:** 1 Department of Molecular Physiology and Biophysics, Vanderbilt University School of Medicine, Nashville, Tennessee, USA.; 2 Division of Endocrinology, Diabetes and Metabolism, Department of Internal Medicine, University of Cincinnati College of Medicine, Cincinnati, Ohio, USA.; 3 Department of Endocrinology, University of Cincinnati College of Medicine and Cincinnati Children’s Hospital Medical Center, Cincinnati, Ohio, USA.

**Keywords:** Metabolism, Glucose metabolism

## Abstract

Several studies have associated the presence of residual insulin secretion capability (also referred to as being C-peptide positive) with lower risk of insulin-induced hypoglycemia in patients with type 1 diabetes (T1D), although the reason is unclear. We tested the hypothesis that C-peptide infusion would enhance glucagon secretion in response to hyperinsulinemia during euglycemic and hypoglycemic conditions in dogs (5 male/4 female). After a 2-hour basal period, an intravenous (IV) infusion of insulin was started, and dextrose was infused to maintain euglycemia for 2 hours. At the same time, an IV infusion of either saline (SAL) or C-peptide (CPEP) was started. After this euglycemic period, the insulin and SAL/CPEP infusions were continued for another 2 hours, but the glucose was allowed to fall to approximately 50 mg/dL. In response to euglycemic-hyperinsulinemia, glucagon secretion decreased in SAL but remained unchanged from the basal period in CPEP condition. During hypoglycemia, glucagon secretion in CPEP was 2 times higher than SAL, and this increased net hepatic glucose output and reduced the amount of exogenous glucose required to maintain glycemia. These data suggest that the presence of C-peptide during IV insulin infusion can preserve glucagon secretion during euglycemia and enhance it during hypoglycemia, which could explain why T1D patients with residual insulin secretion are less susceptible to hypoglycemia.

## Introduction

Types 1 diabetes (T1D) is an autoimmune disease in which most islet β cells are destroyed, leading to insulin deficiency and an inability to maintain blood glucose homeostasis. Since Banting, Macleod, and colleagues first treated T1D patients with insulin 100 years ago, the treatment of the disease has been transformed such that it is manageable with the self-administration of insulin. One of the negative side effects of insulin use, however, is that it can lead to dose overestimations and hypoglycemia. In fact, patient fear of hypoglycemia continues to be a prominent barrier to optimal glycemic control, thereby increasing the risk of developing macro- and microvascular complications (1). Further increasing the risk of iatrogenic hypoglycemia in T1D is that the counterregulatory hormone responses to hypoglycemia are severely diminished in many of these patients (2–6).

Under overnight fasting conditions in healthy adults, the liver produces glucose at a rate of approximately 2.2 mg/kg/min to sustain euglycemia (~90 mg/dL; refs. 7, 8). This rate of production is equal to that of glucose utilization by the rest of the body, approximately 50% to 60% of which is accounted for by the brain (9–12). The precise maintenance of euglycemia is achieved by subtle, minute-to-minute changes in the secretion of glucagon and insulin, which act in opposition to one another to increase or decrease hepatic glucose production (HGP), respectively (13). A portion of the regulation of glucagon secretion has been ascribed to an inhibitory effect of insulin on glucagon secretion, also known as the intraislet hypothesis (14, 15). In fact, the intravenous (IV) infusion of insulin during euglycemia has been shown to lower glucagon levels in dogs (16), healthy humans (17–20), and patients with diabetes (21–23). In contrast, endogenous insulin secretion, such as occurs after a meal, does not have the same inhibitory effect on glucagon levels (24), thereby raising the possibility that endogenous insulin regulates glucagon secretion differently than IV insulin. Numerous clinical studies have shown that T1D patients who have an “insulinogenic reserve,” also referred to as being “C-peptide positive,” experience diminished glucose variability (25–31) and enhanced plasma glucagon responses to insulin-induced hypoglycemia (26–28, 30, 32). We therefore conducted studies to test the hypothesis that coinfusion of C-peptide and insulin would preserve basal glucagon secretion under euglycemic-hyperinsulinemic conditions.

In the event of insulin-induced hypoglycemia in nondiabetic people, formidable counterregulatory hormone responses exist to restore euglycemia. The first is a reduction in endogenous insulin secretion (33), followed by the release of glucagon and then an increase in adrenergic drive if the blood glucose level continues to fall (3, 13, 34–37). Of the latter 2 hormones, glucagon is known to have the higher glycemic threshold for its secretion, thereby making it the first line of defense against a fall in blood glucose, accounting for approximately 70% to 90% of HGP during the first 90 minutes of insulin-induced hypoglycemia (35, 38, 39). Most (31, 40–47), but not all (48), studies have demonstrated that C-peptide positivity confers a protective effect against insulin-induced hypoglycemia. However, although this effect has been ascribed in the past to preserved insulin secretion (25, 27, 28, 30, 41, 45), no studies, to our knowledge, have examined the impact of C-peptide per se on glucagon secretion in response to insulin-induced hypoglycemia. Therefore, we also tested the hypothesis that C-peptide infusion would enhance glucagon secretion and HGP in response to insulin-induced hypoglycemia.

## Results

### Basal period.

**Figure 1:**
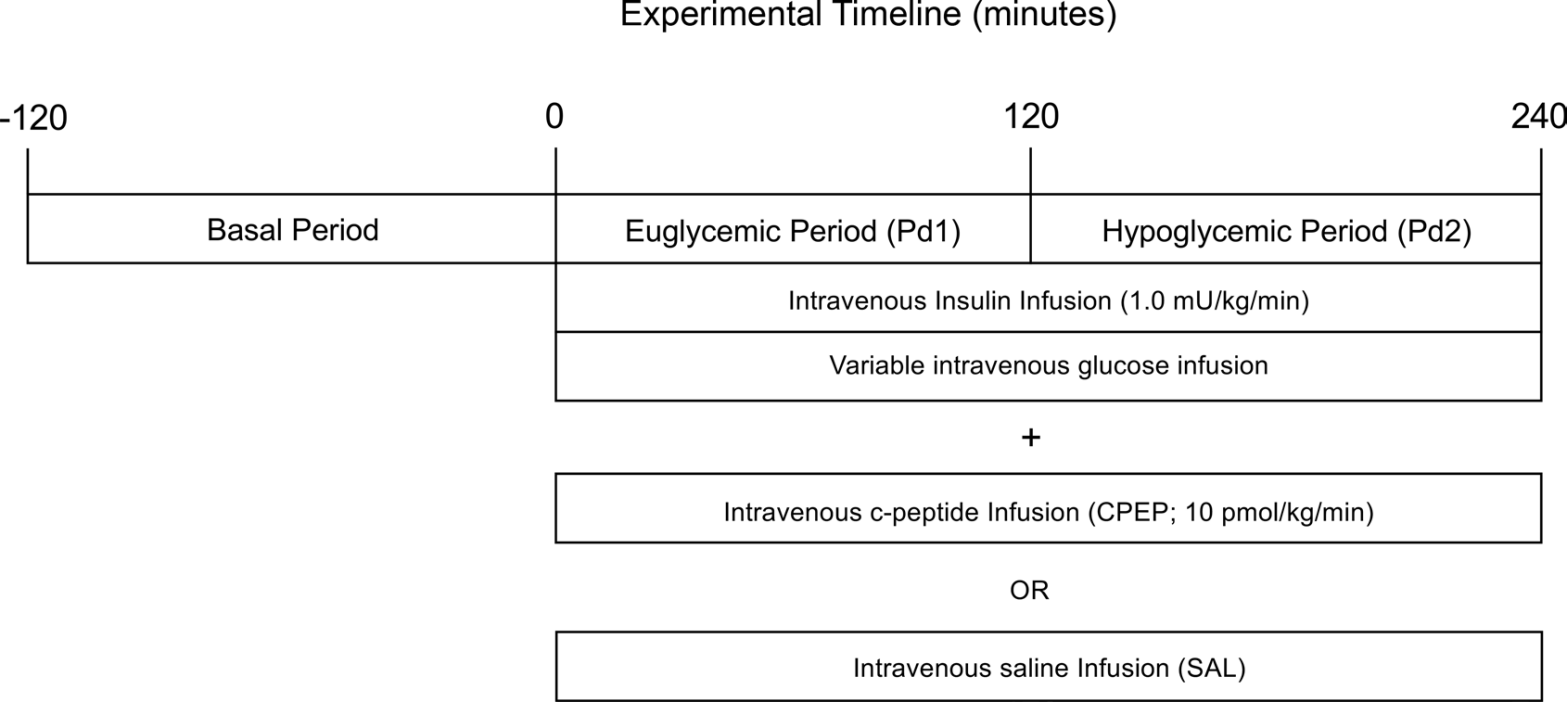
Schematic representation of the metabolic studies.

**Figure 2:**
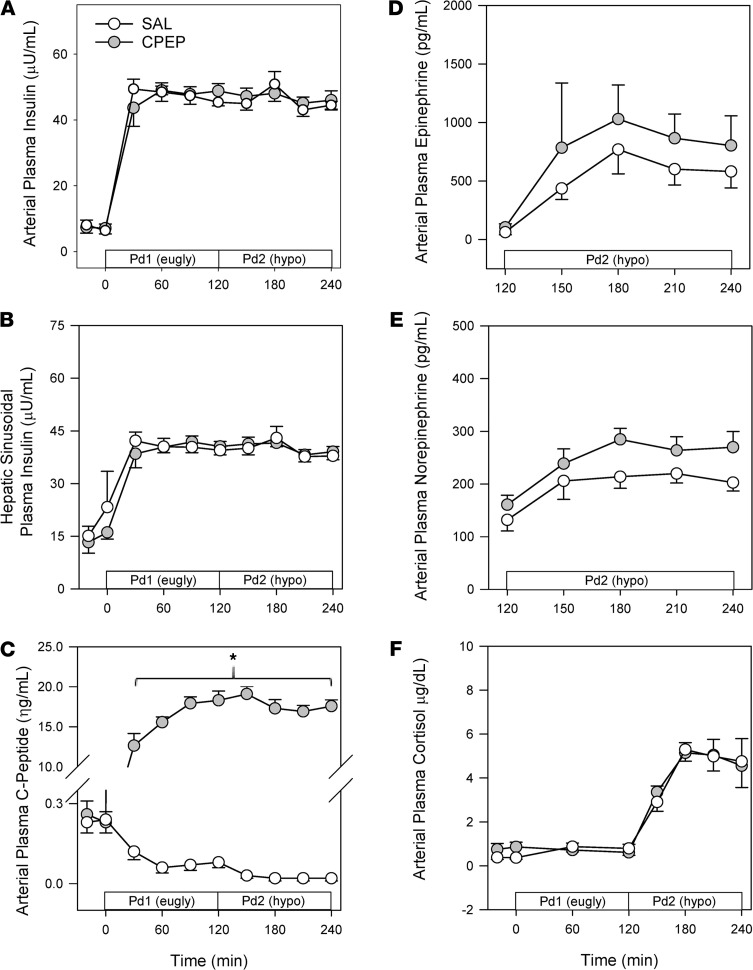
Hormonal responses during the euglycemic (eugly) Pd1 and the hypoglycemic (hypo) Pd2. Arterial insulin (**A**), hepatic sinusoidal insulin (**B**), C-peptide (**C**), epinephrine (**D**), norepinephrine (**E**), and cortisol (**F**). *P < 0.001 between treatments. Data were analyzed using 2-way repeated measures ANOVA. CPEP, C-peptide; Pd1, euglycemic period; Pd2, hypoglycemic period; SAL, saline.

**Figure 3:**
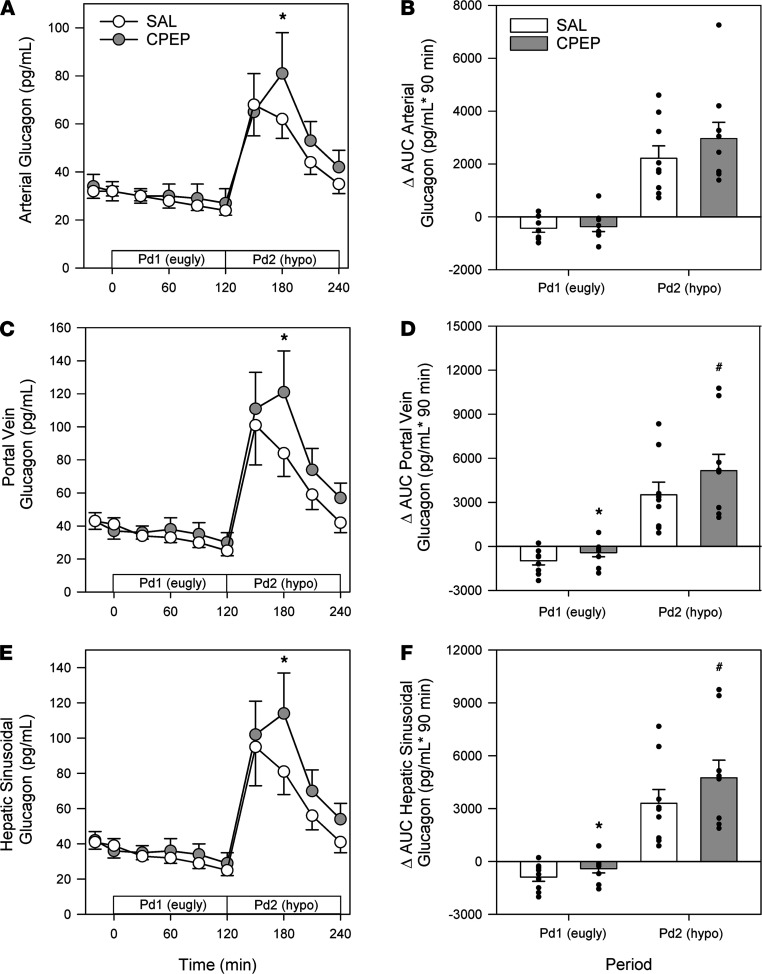
Glucagon responses during the euglycemic Pd1 and the hypoglycemic Pd2. Arterial (**A**), hepatic portal vein (**C**), and hepatic sinusoidal (**E**) plasma glucagon responses during the euglycemic Pd1 and the hypoglycemic Pd2. To the right of these respective graphs (**B**, **D**, and **F**) are the Δ AUC values during the final 90 minutes of Pd1 and Pd2. *P ≤ 0.05 between treatments. ^#^*P* ≤ 0.10 between treatments. Time course data were analyzed using 2-way repeated measures ANOVA. Data for Δ AUC were analyzed using paired 1-way *t* test.

**Figure 4:**
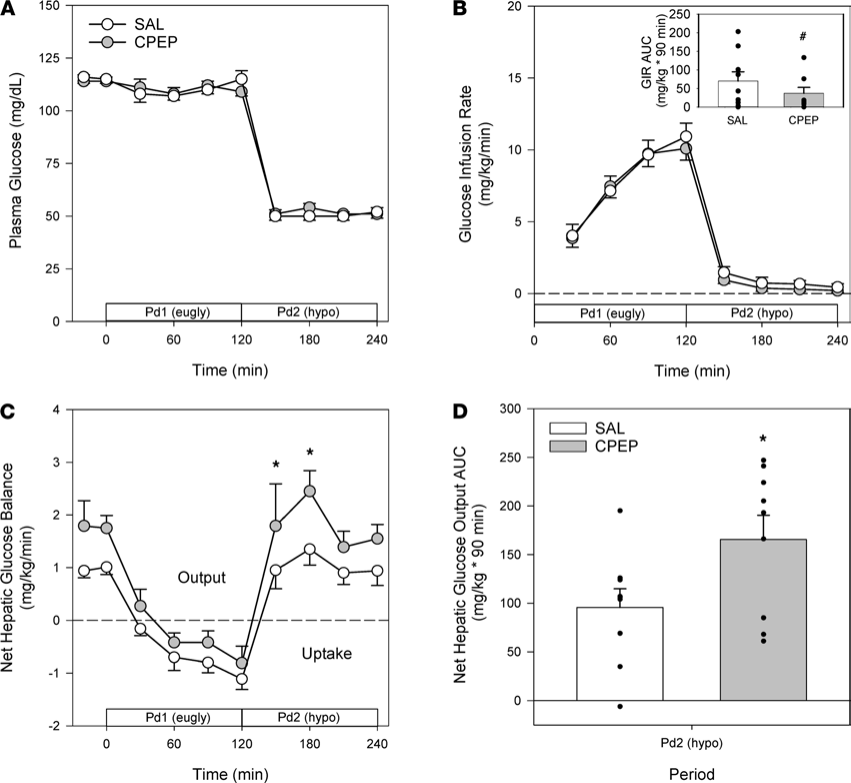
Glucoregulatory responses during the euglycemic Pd1 and the hypoglycemic Pd2. Arterial plasma glucose (**A**), the exogenous glucose infusion rate (GIR; **B**), and the GIR AUC during the final 90 minutes of Pd2 (inset), net hepatic glucose balance (**C**), and AUC for net hepatic glucose balance during the final 90 minutes of Pd2 (**D**). *P ≤ 0.05 between treatments. ^#^*P* = 0.06 between treatments. Time course data were analyzed using 2-way repeated measures ANOVA. AUC data were analyzed using paired 2-way *t* test.

The study design employed can be found in Figure 1. During the basal period (minute –20 to minute 0), plasma levels of insulin, cortisol, and C-peptide were similar between treatments (Figure 2). Likewise, glucagon concentrations were similar in arterial, hepatic portal venous, and hepatic sinusoidal plasma (Figure 3), and net hepatic glucose output and arterial plasma glucose levels were basal (Figure 4). Hepatic blood flow, nonhepatic glucose uptake, and the metabolism of lactate, nonesterified fatty acids (NEFA), and glycerol were indistinguishable between treatment groups during the basal period (Table 1).

### Hyperinsulinemic/euglycemic period Pd1.

The Pd1 insulin infusion (±C-peptide, CPEP) led to a similar increase in insulin concentrations with both treatments (Figure 2, A and B) and a marked increase in C-peptide in the CPEP condition (Figure 2C). Neither hyperinsulinemia nor C-peptide impacted cortisol levels (Figure 2F) or hepatic blood flow (Table 1), and arterial glucose concentrations remained euglycemic (Figure 4A) because of an IV infusion of dextrose that was indistinguishable between treatments (Figure 4B).

**Figure 5:**
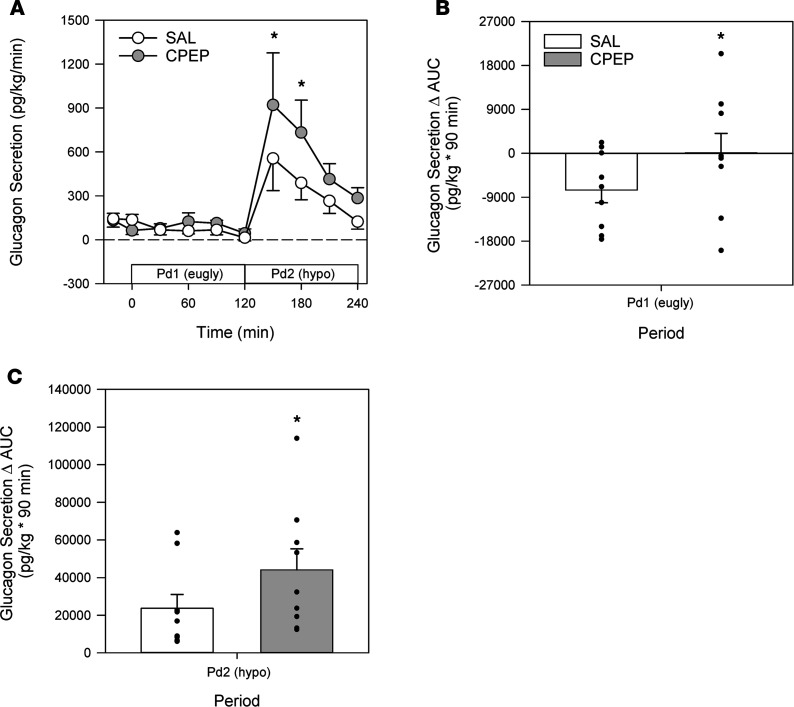
Glucagon secretory responses during the euglycemic Pd1 and during the hypoglycemic Pd2. Glucagon secretion (**A**) and the Δ AUC values (last 90 minutes) for glucagon secretion during Pd1 (**B**) and during Pd2 (**C**). *P ≤ 0.05 between treatments. Time course data were analyzed using 2-way repeated measures ANOVA. Data for Δ AUC were analyzed using paired 2-way *t* test.

Arterial, hepatic portal venous, and hepatic sinusoidal glucagon concentrations were slightly higher in CPEP during the hyperinsulinemic/euglycemic conditions of Pd1, although these differences did not reach significance (Figure 3, A, C, and E). On the other hand, the Δ AUC for portal venous and hepatic sinusoidal glucagon was less in CPEP compared with SAL, meaning that glucagon secretion decreased less in CPEP during Pd1 (Figure 3, D and F). As expected the greater reduction in glucagon in SAL was associated with a greater decrement in glucagon secretion (Figure 5B). In response to hyperinsulinemia, net hepatic glucose output was completely suppressed in both treatments (Figure 4C), in fact leading to slight net hepatic glucose uptake. Because of enhanced glucagon secretion in CPEP and the higher levels of glucagon in the hepatic sinusoids, net hepatic glucose uptake was somewhat lower in CPEP compared with SAL, although this did not reach statistical significance.

In response to the hyperinsulinemia of Pd1, lipolysis, as indicated by plasma NEFA and glycerol levels, was suppressed by approximately 90% in both treatments (Table 1). As a result of diminished availability, net hepatic uptake of these substrates was reduced, with C-peptide having no additional impact (Table 1). Arterial lactate levels were not impacted by hyperinsulinemia, nor were they altered by C-peptide infusion during Pd1 (Table 1).

### Hyperinsulinemic/hypoglycemic period Pd2.

At the start of Pd2 (minute 120), the glucose infusion rate was reduced, and the plasma glucose level was allowed to fall to approximately 51 mg/dL (Figure 4A). In response to insulin-induced hypoglycemia, epinephrine (Figure 2D), norepinephrine (Figure 2E), and cortisol (Figure 2F) levels rose in SAL and CPEP, but there was no significant difference between treatments. As a result of enhanced glucagon secretion (Figure 5, A and C), arterial, portal venous, and hepatic sinusoidal glucagon concentrations rose in both treatments (Figure 3), with the increase being greater with C-peptide infusion. In response to elevated glucagon levels at the liver in CPEP, net HGP during insulin-induced hypoglycemia was 73% higher in CPEP over the final 90 minutes of Pd2 (Figure 4, C and D), leading to a 47% reduction in exogenous glucose required to sustain the plasma glucose at 50 mg/dL (AUC of 70 ± 25 vs. 37 ± 16 mg/kg × 90 minutes in SAL and CPEP, respectively; *P* = 0.06; Figure 4B inset). In response to insulin-induced hypoglycemia, arterial lactate concentrations increased slightly, but similarly, in both treatments, as did net hepatic lactate uptake (Table 1). Increased adrenergic drive to adipose tissue during hypoglycemia caused a sharp rise in NEFA and glycerol levels on each treatment day, which contributed to increased uptake of both by the liver (Table 1), but C-peptide infusion had no impact on lipolysis.

### Between sex analysis.

A total of 5 male and 4 female dogs were studied. Statistical analysis did not reveal any between sex differences during Pd1 or Pd2 for glucagon secretion or net hepatic glucose output.

## Discussion

T1D is a disease that continues to be effectively treated using subcutaneous insulin administration, allowing patients to lead a nearly normal life. Unfortunately, fear of hypoglycemia impedes optimal glycemic control, thereby making these patients more susceptible to developing macro- and microvascular complications. Patients who are C-peptide positive experience debilitating hypoglycemia less frequently than those without an insulinogenic reserve (31, 40–47), although the impact of C-peptide per se on hypoglycemic counterregulation has not been clearly defined. Our data indicate that in response to IV insulin infusion during euglycemia, the coinfusion of C-peptide prevented the expected decline in glucagon secretion. Moreover, C-peptide infusion during insulin-induced hypoglycemia doubled glucagon secretion and increased net hepatic glucose output by 75%, thereby lowering the need for exogenous glucose to maintain glycemia. These data make it evident that C-peptide can play a mitigating role in the suppression of glucagon secretion by insulin, which, if accessed, could lessen the risk of iatrogenic hypoglycemia in insulin-requiring individuals.

Postprandial levels of C-peptide depend on a number of factors, including meal composition and the presence of diabetes. The steady-state arterial value for the CPEP group was 17 ng/mL, which is higher than what is seen in dogs in the postprandial state (~2 ng/mL) but only somewhat higher than what is seen in humans (~10 ng/mL; refs. 49–56), a difference accounted for by a lower clearance rate of C-peptide in humans (~4.4 vs. ~11.6 mL/kg/min; refs. 57–59). On the other hand, the higher peripheral C-peptide levels generated in this study were necessary to more closely mimic the postprandial level seen in the islets by glucagon-secreting α cells. Unlike insulin, C-peptide extraction by the liver is very small, only accounting for approximately 5% of its clearance (56), while most of the remaining fraction is accounted for by the kidneys (60), which argues against a direct effect of C-peptide on hepatic glucose metabolism during both Pd1 and Pd2 of our studies. In addition, the absence of any difference in peripheral glucose metabolism between treatments during Pd2, such as markers of lipolysis and nonhepatic glucose uptake, also supports the conclusion that the higher rates of net hepatic glucose output were a result of hyperglucagonemia and contributed to the diminished need for exogenous glucose. Nevertheless, future studies examining the independent effect of C-peptide on hepatic glucose metabolism and the dose-response relationship between C-peptide and glucagon secretion are required.

There was a nonsignificant increase in adrenergic drive with C-peptide infusion, but the only hormone significantly impacted by C-peptide was glucagon. It has been reported that GPR-146 is the receptor for C-peptide (61) and that these receptors are found in islet α cells (62), thereby making a stimulatory effect plausible. It is also well known that in the absence of C-peptide, IV-delivered insulin lowers glucagon levels in vivo (16–23). Interestingly, C-peptide infusion during Pd1 (euglycemic period) reduced this suppressive effect on glucagon secretion, pointing toward a capability of preserving basal glucagon levels during hyperinsulinemia, which could be of benefit to insulin-requiring individuals between meals or while they sleep. Even more remarkable was that C-peptide’s effect on glucagon secretion was even more pronounced during hypoglycemia, leading to a 2-fold increase in glucagon secretion in the CPEP group compared with SAL, and a 75% increase in net HGP, which decreased the need for exogenous glucose infusion. Although the pathways contributing to increased HGP were not measured (i.e., gluconeogenesis and glycogenolysis), it is likely that enhanced glycogenolysis was the main driver because of glucagon’s known stimulatory impact on this process, especially during the first 90 minutes of insulin-induced hypoglycemia (35, 38, 39, 63).

We are aware of only one other study in which C-peptide was infused to determine its effect on insulin-induced hypoglycemia (64). In that study, Oskarsson and colleagues observed that C-peptide infusion did not impact plasma glucagon levels in patients with T1D who were C-peptide negative. However, there are a number of distinctions between that study and ours that may explain the differing conclusions. Most importantly, our healthy dogs were C-peptide positive, while the 7 T1D patients Oskarsson et al. studied were C-peptide negative, with 2 having “background retinopathy.” It is known from other studies of patients with T1D that glucagon secretion during insulin-induced hypoglycemia is absent in those who are C-peptide negative (27, 28), and that was also the case in the Oskarsson study; there was no rise in glucagon during hypoglycemia whether C-peptide was infused or not. In contrast, glucagon responses to insulin-induced hypoglycemia remain present, but diminished, in C-peptide–positive patients with T1D (26, 28), with the magnitude of this response being proportional to the rise in C-peptide after a mixed-meal tolerance test (30). At first glance, this could imply that the capacity to secrete glucagon diminishes gradually as the T1D patient transitions from being C-peptide positive to C-peptide negative, at which point it ceases to exist. However, we know that α cell “death” in T1D does not occur because the glucagon response to other metabolic stimuli (e.g., arginine) is present (26, 65, 66). When considered in the context of the current results, it is possible that the glucagon responses to insulin-induced hypoglycemia would be proportional to the quantity of residual C-peptide. If this is the case, then the brief exposure to C-peptide (3 hours) after years of it having been absent, and at a venous concentration below what is normally present in the islets after a meal, may not have reached the threshold required to stimulate glucagon secretion in these C-peptide–negative subjects. Finally, it is also noteworthy that limitations of the sampling strategy by Oskarsson and colleagues (because of the use of human patients) rendered them unable to assess the glucagon secretion rate or its levels at the liver (i.e., in hepatic portal vein blood). In fact, while the mean glucagon concentrations in arterial blood were increased with C-peptide infusion in our studies, the increment in the hepatic portal vein, which is the downstream vessel that receives hormone-rich blood from the islets, was even greater. Even with consideration of these methodological differences, our data still point toward C-peptide having an important role in the preservation of glucagon secretion under euglycemic/hyperinsulinemic conditions and its enhancement when the blood sugar falls. Future studies will be required to more closely examine whether these effects of C-peptide on glucagon secretion can be restored in patients who are C-peptide negative and whether chronic replacement of the peptide can stabilize glycemia and/or enhance hypoglycemic counterregulation. In support of this prospect, Johansson and colleagues showed that C-peptide coinfusion with insulin for 1 month improved glycemic control in C-peptide–negative patients with T1D, although hypoglycemic events were not assessed (29).

Given that the current results were attained using the canine model, it remains to be determined if C-peptide coinfusion with insulin has a similar glucagonogenic effect in humans. It should be pointed out, however, that much of what we know about insulin action comes from studies that used the dog model, with the most noteworthy being the work of Banting and Macleod, whose discovery of insulin led to the Nobel Prize in Physiology or Medicine in 1923 (for a review, see ref. 67). Since that time, use of the dog model has continued to provide a portal into how HGP is regulated by the pancreatic hormones because of the remarkable similarity of this interaction to that seen in humans (68). Based on this precedent and the close link between insulin and C-peptide, which exists in the islet β cells from synthesis to secretion, we posit that the translation of our findings to the human is highly likely. Should this be the case, the finding that C-peptide infusion, alongside that of insulin, can enhance glucagon secretion and net hepatic glucose output when the blood sugar is low could have important clinical implications. For example, the coinfusion of C-peptide with insulin could lower the risk of complications in patients with T1D by allowing tighter glycemic control without an accompanying increase in the risk of hypoglycemia. In fact, this applies to all insulin-requiring individuals. While iatrogenic hypoglycemia is most closely associated with the pathology of T1D, it also impedes optimal glycemic regulation in insulin-requiring patients with other diseases, such as type 2 diabetes (69). In addition, after experiencing a bout of hypoglycemia, patients become even more susceptible to developing hypoglycemia because of further diminished counterregulatory hormone secretion, including glucagon (2, 70). It is therefore possible that C-peptide coinfusion with insulin could make these people less vulnerable to low blood sugar in the wake of a previous hypoglycemic event.

In summary, our data demonstrate that IV-infused C-peptide preserves basal glucagon secretion during euglycemic-hyperinsulinemia in dogs, thereby suggesting that it plays a role in intraislet signaling and the in vivo regulation of glucagon secretion. Moreover, C-peptide infusion also doubled glucagon secretion in these animals during insulin-induced hypoglycemia, which increased net HGP by 75% and lowered the need for exogenous glucose infusion. These data support the hypothesis that C-peptide–positive T1D patients may be less susceptible to insulin-induced hypoglycemia because of enhanced counterregulatory responses conferred by C-peptide. Future studies will be required to determine the translational significance of this finding in patients with diabetes, as it could lead to novel therapies that lower the incidence of hypoglycemia and improve glycemic control in insulin-treated patients, thereby mitigating their risk of developing micro- and macrovascular complications.

## Methods

### Animal care, diet, timeline, and surgical procedures.

Studies were carried out on 18-hour fasted adult mongrel dogs (21 ± 4 kg; mean ± SD; 5 male, 4 female), aged 12.1 ± 0.6 months and acquired from a US Department of Agriculture–approved vendor. The animals were housed in a facility with a 12-hour light/12-hour dark cycle (lights on at 06:00 hours) and fed once daily a standard chow and meat diet (34% protein, 14.5% fat, 46% carbohydrate, and 5.5% fiber based on dry weight) that was weight maintaining (53).

Two weeks prior to being studied, each dog underwent surgery under general anesthesia to insert sampling catheters in a femoral artery, the hepatic portal vein, and the left common hepatic vein and to place blood flow probes (Transonic Systems) around the hepatic portal vein and hepatic artery (53). Two experiments were conducted on each animal, with the first being approximately 15 days after surgery and the second approximately 15 days later. Two days before each experiment, blood was drawn to measure leukocyte and hematocrit counts for each animal. Animals were only studied if they had a leukocyte count less than 16,000/mm^3^, a hematocrit more than 35%, a good appetite (noted by the consumption of at least 600 of the 800-calorie daily ration), and normal stools. On the morning of each experiment, the sampling catheters and flow probes were removed from their subcutaneous pockets under local anesthesia (2% lidocaine, Hospira), and the animal was placed in a Pavlov harness. Intravenous catheters were then inserted into a cephalic and saphenous vein to allow peripheral infusions as necessary. The animal was then allowed to rest for 100 minutes, after which basal samples were collected from the artery and portal and hepatic veins between –20 and 0 minutes.

After the 0-minute sample was collected, experimental period 1 (Pd1) began with the infusion of insulin at 1 mU/kg/min into a leg vein (Figure 1). At the same time, a leg vein infusion of either canine CPEP (10 pmol/kg/min; AnaSpec) or SAL was also started, and dextrose was infused as needed to maintain euglycemia throughout Pd1 (0–120 minutes). Studies were randomized such that 4 of the 9 dogs received the C-peptide on the first study and the other 5 received saline first. At minute 120, the dextrose infusion was reduced, and the plasma glucose level was allowed to fall to approximately 50 mg/dL, where it was clamped (Pd2; 120–240 minutes; Figure 1). At the end of the first experiment, all infusions were halted with the exception of glucose, which was infused as needed to restore euglycemia. Once that infusion was no longer required, the catheters and flow probes were placed back into subcutaneous pockets under general anesthesia. After the second experiment, animals were euthanized with pentobarbital, the abdomen was opened, and the positions of the catheter tips were verified.

### Specimen analyses.

The processing of blood samples has been described previously (71). Plasma glucose was analyzed using the glucose oxidase method (Analox Instruments). Insulin, glucagon, and C-peptide were measured using commercially available radioimmunoassay (RIA) kits from MilliporeSigma, and cortisol was measured using an RIA protocol developed by the Vanderbilt University Medical Center Hormone Assay & Analytical Services Core using an in-house antibody (gift from W. Nicholson) and ^125^I-cortisol (MP Biomedicals). Catecholamines were measured using high-performance liquid chromatography, while NEFA (FUJIFILM Medical Systems) and lactate and glycerol concentrations (72) were measured using fluorometric assays (73).

### Calculations and data analysis.

Hepatic blood flow was measured using ultrasonic flow probes (Transonic Systems). Net hepatic glucose balance (NHGB), hepatic sinusoidal insulin and glucagon levels, and nonhepatic glucose uptake were calculated as described previously (74), while plasma glucose levels were converted to whole blood values for the calculation of NHGB (75, 76). Glucagon was measured every 30 minutes during Pd1 and Pd2, and those data are expressed as absolute values and as Δ AUC. The Δ AUC was calculated over the final 90 minutes of Pd1 and Pd2 as follows: ([AvgGGN_t0–30_ – BslGGN] × 30min), where AvgGGN_t0–30_ refers to the average glucagon level over a given 30-minute period, BslGGN refers to the average glucagon level during the basal period (i.e., the average of the –20-minute and 0-minute time points), and 30min represents the 30-minute sampling period. Three 30-minute calculations were then summed to provide the final number in units of pg/mL × 90min. Glucagon secretion was calculated by multiplying the difference in plasma glucagon levels between the hepatic portal vein and artery by plasma flow in the hepatic portal vein.

### Statistics.

All data are presented as mean ± SEM unless stated otherwise. Statistical analyses were carried out using SigmaStat software (Aspire Software International). Time course data were analyzed using 2-way ANOVA with repeated measures, and post hoc comparisons were made as appropriate. AUC data in Figure 3 were analyzed using paired 1-way *t* test and in Figure 4 and Figure 5 were analyzed using paired 2-way *t* test. Significance was set at *P* < 0.05.

### Study approval.

The protocol was approved by the Vanderbilt University IACUC, and the animals were housed and cared for according to Association for Assessment and Accreditation of Laboratory Animal Care International guidelines.

## Author contributions

MCM, MS, BF, and JJW performed the studies. MCM, SOW, YD, NS, ADC, and JJW wrote the manuscript. RLC prepared the figures. MCM, ADC, and JJW designed the experiments and analyzed the data. JJW received funding to conduct the studies.

## Figures and Tables

**Table 1:**
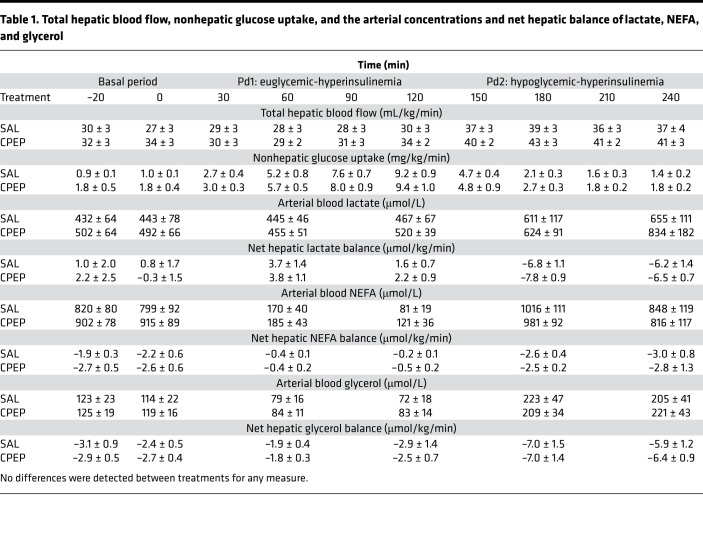
Total hepatic blood flow, nonhepatic glucose uptake, and the arterial concentrations and net hepatic balance of lactate, NEFA, and glycerol

## Version 1 05/18/2021

In-Press Preview

## Version 2 06/22/2021

Electronic publication
